# The algorithmic epistemic inversion in gastroenterology: how social media and psychological vulnerability fuel secondary risks in IBD and IBS management

**DOI:** 10.3389/fpubh.2026.1871219

**Published:** 2026-08-19

**Authors:** Li An, Mingchun Luan, Yongping Yang, Siping Wang

**Affiliations:** Yantaishan Hospital, Yantai, China

**Keywords:** algorithmic epistemic inversion, Brain-Gut-Algorithm Axis, IBD, IBS, medical misinformation, psychogastroenterology

## Abstract

The unchecked proliferation of medical misinformation on short-video platforms has crystallized into a critical digital public health crisis. For patients managing chronic gastrointestinal diseases such as IBD and IBS, recommendation algorithms routinely override evidence-based medicine (EBM), engendering a phenomenon we term “algorithmic epistemic inversion.” This inversion amplifies unproven “miracle cures” and fear-based narratives, driving population-level secondary risks, ranging from indiscriminate probiotic abuse and extreme elimination diets, malnutrition, and orthorexia nervosa. Although IBD and IBS are primarily gastrointestinal diseases, these algorithmically induced secondary risks impose severe, systemic damage on the central nervous system (CNS) and broader neurocognitive functions. To conceptualize this crisis, we introduce the novel framework of the Brain-Gut-Algorithm Axis, which delineates how algorithm-amplified anxiety acts as a social contagion exacerbating somatic symptoms through established gut–brain pathways. To counter this infodemic, we advocate for a public health governance framework grounded in AI-driven algorithmic pharmacovigilance. We urge policymakers and clinicians to recognize algorithm hegemony as a critical digital determinant of health by advancing health equity and integrating psychogastroenterology into routine clinical practice. This Perspective provides specific regulatory and clinical strategies that are crucial to protecting vulnerable populations and strengthening digital public health governance.

## 1 Introduction

The Epistemic Inversion and Algorithmic Hegemony: Misinformation on social media has become an urgent digital health crisis, particularly for individuals managing chronic gastrointestinal diseases (1, 2). For patients navigating the relapsing–remitting course of IBD, platforms like TikTok, YouTube, and Instagram have become the primary therapeutic consultancies (3, 4). The clinical implications of this shift extend beyond mere symptoms and have a profound effect on patients’ lives; research indicates that algorithm-driven probiotic fads can cause serious, unintended physiological and neurocognitive complications (5). The extent of this impact is vast. For instance, consumer-generated content constitutes the overwhelming majority of health hashtags on TikTok, with healthcare professionals contributing merely 1%, creating a massive information vacuum where peer narratives far outweigh evidence-based clinical recommendations (6). This dominance is further reflected in engagement metrics. A recent cross-sectional study of over 62 million cumulative views across 319 YouTube videos on gut microbiome demonstrated a stark inverse relationship between information quality and public engagement. Videos from non-medical independent creators and patients achieved significantly higher daily and total viewership compared to those made by verified medical professionals. This disconnect reflects a pronounced ‘comfort gap,’ wherein patients exhibit greater affinity for peer-led anecdotes and demonstrably lower-quality content than with high-scoring, formal clinical guidance (7, 8). This gap is exploited by a perilous “eminence-based practice,” which influencers without formal training are considered unquestionable medical authorities simply because of their large follower counts and polished digital presence (9). A recent thematic analysis of IBS-tagged TikTok videos specifically tagged with IBS highlights the severity of this issue: almost half of the content originates from influencers, and a striking 86% of the analyzed videos fail to provide factual educational information. Despite profound deficit in clinical actionability and understandability, influencer content is shared significantly more likely than evidence-based videos from healthcare providers, actively fueling the viral spread of misinformation (10).

The clinical reality of such a shift is stark. Surveys indicate that up to 94.2% of health practitioners routinely see patients presenting with health misinformation, demonstrating that the digital echo chamber has infiltrated the clinical consultation room (11). Given the global burden of these diseases being high (perceived food intolerances affect up to 20% of the general population), this self-diagnosed cohort represents a massive market for digital content providing simplistic, non-immunological explanations for GI distress (12). This algorithmic hegemony further engenders a critical health equity crisis, as a novel “digital determinant of health.” Marginalized populations, frequently facing structural barriers to specialized gastroenterological care, disproportionately rely on free social media platforms for medical guidance. Consequently, algorithmically amplified misinformation disproportionately targets these vulnerable groups. Recommendation engines prioritizing engagement over evidence-based medicine (EBM) not only propagate falsehoods but also exacerbate existing health disparities by directing socioeconomically disadvantaged patients into unregulated, costly, and potentially hazardous alternative interventions (13). This digital determinant is acutely magnified in Low- and Middle-Income Countries (LMICs). In regions where gastroenterological specialists are scarce yet mobile internet penetration is ubiquitous, social media frequently serves as the absolute primary source of medical care. Thus the algorithmic amplification of misinformation disproportionately exploits the Global South, exporting Western dietary fads and unverified supplement markets to populations with minimal regulatory protection and structural clinical support. The persistence of this infodemic raises a fundamental regulatory question: why does this misinformation remain largely unregulated, and who bears responsibility for policing it? Currently, a profound regulatory vacuum persists. In many jurisdictions, legal frameworks such as Section 230 of the U.S. Communications Decency Act shield social media companies from liability for user-generated content, treating them merely as neutral conduits rather than active publishers. Without the legal imperative to aggressively police medical claims, platform governance remains entirely contingent on corporate self-regulation. Furthermore, these platforms are algorithms explicitly designed to maximize user retention and engagement. Because human cognition is inherently drawn to headline-grabbing, polarizing, or emotionally charged narratives, these algorithms inadvertently amplify sensationalized “miracle cures” and extreme dietary dogmas over nuanced, factual EBM (14). This engenders a structural conflict of interest: the very mechanisms that drive platform profitability actively undermine public health.

While democratization of medical knowledge is inherently valuable, the current digital ecosystem is currently functioning solely within an “attention economy” (15). As cogently highlighted by the Lancet and Financial Times Commission, business models based on aggressive data extraction and viral dissemination of misinformation are actively widening existing health disparities (16). The digital translation of EBM is exceedingly poor: a staggering 97% of patient-generated videos and 65% of professional-generated videos fail to cite any peer-reviewed scientific evidence supporting their dietary claims (7). This pervasive lack of scientific rigor exacerbates an “epistemic inversion”—presenting unsupported, low-quality observations as clinical truths (17). Recommendation algorithms, optimized for engagement, systematically suppress nuanced clinical advice while proliferating emotionally charged micro-narratives, defining the current infodemic (6, 11, 18). Addressing this epistemic inversion requires a departure from the traditional, atomized physician-patient paradigm. The unregulated viral dissemination of dietary dogmas must now be recognized as a pressing public health policy challenge, directly threatening population health literacy. The management of medical misinformation on short-video platforms can no longer depend exclusively on reactive clinical corrections or individual eHealth literacy. Instead, it necessitates structural, macro-level interventions to mitigate the systemic public health risks posed by engagement-driven algorithms.

## 2 Secondary clinical risks: from probiotic abuse to malnutrition

Algorithmic amplification of unregulated “gut health” content has direct translation into tangible secondary clinical harms. While localized evidence highlights severe impacts—such as 85% of patients resolving brain fogginess upon discontinuing unverified supplements (5)—the precise population-level proportion of individuals harmed by this misinformation remains unquantified. This critical data gap underscores an urgent need for future large-scale epidemiological studies to precisely map the scope of these algorithm-induced risks.

### 2.1 Public health hazards of unregulated probiotic overuse

Social media algorithms frequently frame “gut dysbiosis” as the universal etiology of modern ailments, driving a lucrative market for dietary supplements. This narrative fundamentally disregards a core scientific reality: defining a universal ‘normal’ microbiota remains elusive due to immense inter-individual variation (19). Furthermore, the algorithmic narrative obscures well-established environmental drivers of bona fide dysbiosis. A sustained “Westernized diet” directly depletes protective, short-chain fatty acid (SCFA)-producing bacteria while promoting pro-inflammatory phyla. By obscuring this complex diet-microbiome interaction, algorithms absolve users from dietary responsibility, falsely marketing unregulated probiotic supplements as a quick remedy for lifestyle-induced inflammation (20).

This reductionism extends to clinical language. While influencers conflate all gaseous distension with ‘SIBO,’ clinical guidelines mandate precise definitions, such as classifying methane-producing organisms as Archaea (Intestinal Methanogen Overgrowth, IMO) (21). The multi-billion-dollar supplement industry, largely unregulated, frequently evades clinical validation. Empirical data substantiates this commercial bias: analysis of top-viewed gut microbiome videos reveals that a substantial portion promote health-related interventions or dietary products for financial gain, frequently without disclosing conflicts of interest. Crucially, videos containing these promotional materials score significantly lower on validated scientific quality indices (such as the DISCERN criteria) than non-promotional content (8). For instance, while increasing dietary omega-3 fatty acids from marine fish is prudent for ulcerative colitis (UC), omega-3 supplements are explicitly not recommended owing to a lack of proven efficacy (22). Consequently, the AGA guidelines recommend probiotics for Crohn’s disease, UC, and IBS only within the context of clinical trials (23). Comprehensive reviews indicate that probiotics are largely ineffective for inducing or maintaining remission in Crohn’s disease, and algorithms routinely omit the practical clinical realities of adverse effects that lead to significant patient discontinuation rates (24).

In practice, the indiscriminate, population-level introduction of bacterial loads in individuals with underlying dysmotility frequently precipitates Small Intestinal Bacterial Overgrowth (SIBO). Rather than focusing narrowly on micro-biological mechanisms—such as specific metabolic toxicities associated from unmonitored bacterial fermentation—public health officials must recognize the epidemiological magnitude of this risk. Mass probiotic overuse has evolved into an epidemiological public health risk, escalating systemic healthcare costs and inducing population-level malnutrition. Algorithmic marketing for profit drives the mass consumption of unverified probiotics, bypassing regulatory clinical pathways and creating a widespread, unchecked public health hazard (13). Beyond physiological harm, this algorithmic pipeline engenders profound “financial toxicity”: vulnerable patients routinely exhaust out-of-pocket resources on predatory, high-cost supplements devoid of empirical backing, while public healthcare systems bear the downstream economic burden of treating algorithmic-induced complications, including severe SIBO exacerbations, delayed evidence-based treatment, and hospitalization for diet-induced malnutrition. Characterizing algorithmic misinformation as merely a clinical communication problem obscures its substantial systemic economic impact, necessitating public health interventions that hold digital platforms liable for the economic externalities generated by their recommendation engines.

### 2.2 Population-level malnutrition and disordered eating from extreme elimination diets

Simultaneously, algorithms aggressively promote restrictive dietary regimens—rebranding them as miraculous “gut detoxes” (12). Influencers frequently misinterpret rapid symptomatic shifts as clinical success; however, short-term dietary changes induce immediate shifts in bacterial populations that are a *consequence* of the intervention rather than the *cause* of disease remission (18). While clinical evidence confirms that drastic, unmonitored dietary shifts can induce secondary states of dysbiosis and severe mucosal barrier degradation, the primary public health concern lies in the population-level scale of this phenomenon. At a population level, the viral propagation of extreme elimination diets functions as a social contagion. The normalization and incentivization of “diet-stacking,” a high-risk behavior whereby individuals progressively accumulate increasing numbers of dietary restrictions without guidance from professionals, turns dietary management from a niche clinical tool into a widespread vector for systemic malnutrition. True EBM demands phenotype-specific dietary tailoring, a nuance entirely lost on digital platforms. For example, IOIBD guidelines recommend moderate-to-high intake of fruits and vegetables in Crohn’s Disease to promote SCFA production, restricting insoluble fibers only in patients with symptomatic fibrostricturing disease. Recommendation algorithms collapse these guidelines into universal, restrictive dogmas, predisposing patients to nutritional deficits and an irrational fear of food (7, 22). In IBD patients with compromised mucosal absorption, extreme elimination diets precipitate severe malnutrition, sarcopenia and mucosal barrier degradation (22, 24). This culminates in ‘diet-stacking’—a high-risk behavior wherein patients progressively adopt multiple dietary restrictions without professional supervision (12). Systematic reviews confirm that individuals with dietary-controlled GI disorders inherently exhibit a significantly higher prevalence of disordered eating practices. Algorithms directly exploit this baseline vulnerability by normalizing and rewarding extreme food restriction (25). Given that patients with active IBD are at a structurally heightened risk for protein-calorie malnutrition, evidence-based practice mandates that restrictive interventions must be strictly accompanied by specialized nutritionists—a safety net that algorithms entirely bypass (20, 24).

## 3 The psychological vortex: introducing the “Brain-Gut-Algorithm Axis”

**Figure 1:**
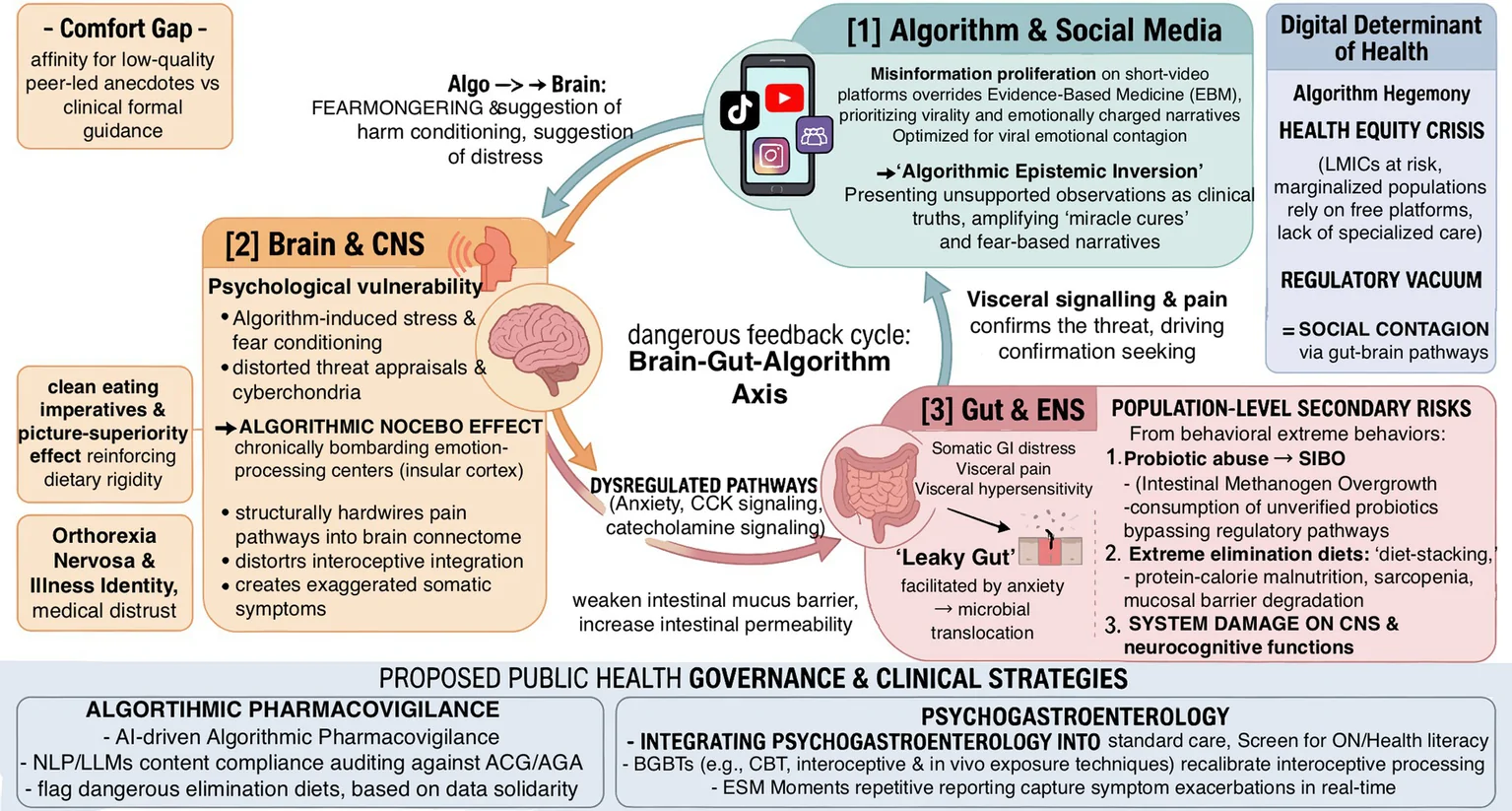
The Brain-Gut-Algorithm Axis. This infographic illustrates the closed feedback loop among social media algorithms, psychological vulnerability, and gut health. It delineates algorithm-triggered stress, its secondary clinical manifestations (e.g., SIBO) and associated risk behaviors (e.g., extreme diets), and proposes AI-enabled pharmacovigilance and integrated psychogastroenterology practice as public health intervention strategies.

Bidirectional communication between the central and enteric nervous systems is a well-established physiological pathway. This framework is validated by the Rome IV consensus, which formally redefined Functional Gastrointestinal Disorders (FGIDs) as “Disorders of Gut-Brain Interaction,” establishing that GI symptoms arise from a complex interplay of central nervous system (CNS) dysregulation and altered gut signaling, even absent structural tissue damage (26). Within this framework, external psychological stressors—such as algorithmic fearmongering—are primary modulators that can directly disrupt gut motor function. Psychological stress triggers catecholamine signaling that weakens the intestinal mucus barrier, increases epithelial permeability, and facilitates microbial translocation. The “leaky gut” promoted by digital influencers is therefore often a downstream direct physiological consequence of the same anxiety their content induces (27). This human-amplification creates a dangerous feedback cycle we term the “Brain-Gut-Algorithm Axis” (Figure 1), wherein digital fearmongering leverages psychological vulnerability to trigger somatic GI distress (7).

### 3.1 Algorithmic nocebo effect: a public health driver of exaggerated somatic symptoms

Social media thrives on demonizing conventional food groups to capture user attention (9). Clinical research demonstrates that even unintended negative suggestions from healthcare professionals can reliably trigger nocebo responses. Social media algorithms dangerously amplify this vulnerability by intentionally weaponizing fear-based rhetoric to maximize user retention (27). Patients exposed to these threats develop profound food anxieties owing to the extreme sensitivity of the brain-gut axis. The intense psychological expectation of harm triggers a potent nocebo effect even when ingesting objectively harmless foods (28). Algorithm-induced stress effectively amplifies normal gut signaling into hyper-perceived visceral pain (26). Experimental nocebo research reveals that this response is characterized by a marked decrease in dopamine and endogenous opioids alongside an increase in cholecystokinin (CCK), which directly amplifies pain pathways. When algorithms chronically bombard emotion-processing centers (e.g., the insular cortex) with threat-based narratives, they actively distort interoceptive integration, structurally hardwiring the nocebo effect into the brain connectome (27, 28).

### 3.2 Orthorexia nervosa and “illness identity”

Trapped within algorithmic echo chambers, patients are subjected to a continuous barrage of ‘clean eating’ imperatives that normalize pathological dietary rigidity (3). This environment transforms the quest for symptom relief into Orthorexia Nervosa (ON) a clinically significant obsession with dietary purity. This obsession intertwines with the severe anxiety GI patients experience regarding potential food cross-contamination, which algorithms amplify into a generalized, pathological avoidance of all unverified foods (25). Empirically, this psychological obsession is linked to specific digital architectures. The frequency of Instagram use is positively correlated with orthorexic symptoms severity, primarily attributable to the “picture-superiority effect,” whereby curated, image-heavy feeds reinforce dietary rigidity more effectively than text (9). This algorithmic phenomenon is now recognized as a widespread clinical risk. A recent systematic scoping review of 31 studies confirmed a bidirectional relationship between ON and social media use, demonstrating that orthorexic symptoms is directly amplified by three distinct algorithmic variables: the use of highly visual platforms (e.g., Instagram, TikTok), extended daily screen time, and targeted exposure to fitness and “clean eating” content. Furthermore, this digital-to-somatic harm pathway disproportionately affects specific demographic groups, with female users, adolescents, and young adults who exhibit heightened vulnerabilities due to algorithmic exploitation of baseline body dissatisfaction and health anxiety (29). This algorithmic targeting is particularly devastating for pediatric and adolescent populations. Adolescence represents a critical window for neurocognitive development and identity formation, perfectly overlapping with the peak onset of many chronic GI conditions. When algorithms channel pediatric and adolescent populations, groups which inherently represent low health literacy groups with heightened susceptibility to peer influence, into these dietary echo chambers, the resulting orthorexia and malnutrition carry severe, long-term developmental consequences. Urgent targeted public health measures are necessary to specifically curtail the algorithmic dissemination of unverified restrictive diets to minors, shielding pediatric GI patients from digital epistemic inversion. Additionally, alternative therapy communities leverage patients’ prior medical trauma, fostering a potent “illness identity” that breeds entrenched medical distrust. This phenomenon is particularly concerning, given that early life adversity and trauma are established neurobiological vulnerabilities in GI disorders, known to cause persistent neuroendocrine dysregulation and heightened stress responses. Recommendation algorithms systematically exploit this baseline hyperarousal and trauma-induced hypervigilance, trapping the most psychologically vulnerable patients in echo chambers that validate their distress while bypassing formal, evidence-based somatic trauma interventions (30). Peer-to-peer validation on these platforms bypasses formal medical intervention, rendering patients less amenable to objective clinical de-escalation (6).

## 4 Future directions: algorithmic pharmacovigilance and psychogastroenterology

Addressing the clinical fallout of the Brain-Gut-Algorithm Axis requires a paradigm shift integrating macro-level digital governance with micro-level clinical adaptation.

### 4.1 Establishing AI-driven algorithmic pharmacovigilance

Because vulnerable patients cannot individually outmaneuver engagement-driven algorithms, the global public health community must advocate for structural platform accountability through ‘Algorithmic Pharmacovigilance’. This concept aligns directly with the World Health Organization’s (WHO) calls for robust infodemic management and must be legally anchored in emerging frameworks such as the European Union’s Digital Services Act (DSA), which mandates that enormous online platforms mitigate systemic risks to public health. Grounded in the principle of “data solidarity,” this model demands a departure from inadequate, individual-level data control. Instead, corporate practices that prioritize viral medical misinformation over clinical safety must be classified as public health hazards, subject to severe, profit-deterring regulatory fines enforced by supranational harm mitigation bodies (1, 16). Currently, platform accountability is severely hindered by the “black box problem.” Algorithms are zealously guarded as proprietary trade secrets and mathematical formulas, precluding independent health researchers from auditing how medical misinformation is prioritized and distributed. While tech platforms employ their own Natural Language Processing (NLP) algorithms for content moderation, these automated systems struggle to comprehend the scientific context and nuances of gastrointestinal health. Furthermore, recent reductions in human trust and safety teams across the tech sector have left algorithms to moderate complex medical speech without necessary human oversight (14).

We propose implementing Content Compliance Auditing frameworks with Natural Language Processing (NLP) and Large Language Models (LLMs) to act as AI auditors that can automatically parse viral GI-related videos and cross-reference claims against EBM guidelines like AGA/ACG criteria to flag dangerous elimination diets (23). By applying specific criteria for downgrading evidence quality as identified by GRADE, AI can systematically categorize anecdotal claims as “very low quality” evidence, enabling transparent evaluation and relieving acute administrative pressure on frontline gastroenterologists (11, 17). The implementation of AI-driven algorithmic pharmacovigilance extends beyond individual clinical triage; it constitutes a critical public health surveillance infrastructure. By systematically monitoring short-video platforms for the viral spread of unregulated GI interventions, public health authorities can issue targeted digital counter-narratives and mandate algorithmic down-ranking of unverified medical claims. This proactive public health framework shifts the burden of harm prevention from the individual patient back to platform governance. Crucially, the success of this public health infrastructure depends on legislative mandates compelling tech conglomerates to provide independent researchers with unfettered API (Application Programming Interface) data access. Without transparent access to black-box algorithmic pathways, public health agencies cannot accurately map the epidemiological spread of dietary misinformation or rapidly deploy EBM-backed digital counter-narratives to disrupt the epistemic inversion loop (13).

### 4.2 Integrating psychogastroenterology into mainstream practice

Gastroenterologists can no longer focus solely on the gastrointestinal system; they must actively address the psychological trauma affecting the brain. Integrating psychogastroenterology into standard care is essential (31). Clinicians must utilize rapid screening tools to assess digital health literacy and algorithm-induced eating disorders. Moreover, given that standard retrospective questionnaires are subject to recall and ecological bias, evaluating the real-time somatic impact of algorithmic fearmongering necessitates upgraded clinical endpoints. Clinicians should consider employing the Experience Sampling Method (ESM) via electronic momentary repetitive recordings. This digital approach captures symptom exacerbations in real-time, providing highly sensitive insights into how specific social media interactions immediately trigger gut-brain dysregulation in daily life (32). Cognitive Behavioral Therapy (CBT) must be deployed as a primary treatment. Based on recent epidemiological syntheses, these screening protocols must explicitly capture patients’ specific social media habits, including daily usage duration and primary platform engagement—as these metrics are critical predictors of ON severity (29). Furthermore, social media literacy training should be integrated alongside Brain-Gut Behavior Therapies (BGBTs)—an umbrella term recently endorsed by the Rome Foundation for non-pharmacologic interventions that specifically remediate gut-brain dysregulation. Framing psychological care explicitly as BGBTs reduces patient stigma, validates the biological reality of their symptoms, and increases acceptance of integrated care (30). Within the BGBT framework, Cognitive Behavioral Therapy (CBT) must be deployed as a primary treatment. By targeting faulty threat appraisals, CBT successfully recalibrates dysregulated interoceptive processing and induces measurable neuroplastic changes, directly neutralizing the algorithmic trauma (27). Specifically, social media fearmongering operates as a powerful engine for ‘fear conditioning,’ teaching vulnerable patients to associate benign visceral sensations with catastrophic medical outcomes. To effectively counter this, BGBTs must employ targeted interoceptive and *in vivo* exposure techniques. These evidence-based behavioral experiments allow patients to systematically unlearn maladaptive dietary avoidance and re-evaluate their algorithmically exaggerated visceral anxiety (30).

Clinicians must prioritize de-escalating unverified supplement regimens—a step proven to resolve brain fogginess in 85% of cases (5)—and guide patients toward sustainable, anti-inflammatory dietary patterns, such as the Mediterranean diet. Given that IBS patients prefer dietary interventions (48%) over drugs (29%) or psychotherapy (23%), algorithms frequently drive them toward unverified digital fads. To counter this, clinicians should reframe evidence-based psychotropics as ‘gut-brain neuromodulators’ rather than ‘antidepressants’ to mitigate stigma. Explicitly articulating their physiological roles in regulating pain receptors and motility effectively enhances treatment adherence and diminishes the influence of algorithmic misinformation (32). Psychogastroenterological assessments must carefully distinguish between healthy clinical dietary compliance and algorithm-amplified, anxiety-driven restriction that masquerades as diligence (25). Research confirms that ON is fueled by an obsession with the perceived “purity” of food; for GI patients experiencing a loss of bodily control, the strict rules promulgated by influencers serve as a maladaptive coping mechanism requiring early intervention (9).

Finally, exclusive reliance on individual Health literacy to counter algorithmic hegemony is structurally flawed. The principles of data solidarity dictate that societies bear a collective responsibility to ensure digital practices improve lives, thereby shifting the primary burden of harm prevention away from the overwhelmed patient (16, 18). To counteract pathogenic narratives, clinical communication must strategically emphasize treatment tolerability and utilize “permitted non-information” for mild adverse events to avoid triggering unnecessary nocebo responses. By evolving from passive observers into active digital health creators, clinicians can harness the power of words to heal, effectively neutralizing the digital fearmongering that pervades the algorithmic echo chamber (28). To maximize dissemination and counter the virality of unverified content public health strategies should also foster strategic partnerships between healthcare organizations and established digital influencers. Integrating the highly shareable, engaging formats of influencer content with the rigorous understandability and actionability of evidence-based medical advice can effectively bridge the digital health literacy gap (10). Beyond the clinic setting, mitigating this crisis demands large-scale public health education interventions. Public health authorities must develop and implement population-level digital health literacy campaigns that teach critical appraisal of algorithmic content. Rather than relying on gastroenterologists to counter misinformation within isolated 15-min consultations, these macro-level campaigns should directly target the demographics most susceptible to social media medical misinformation, fostering collective resilience against digital fearmongering.

## 5 Conclusion

This Perspective identifies algorithmic epistemic inversion as a global public health crisis and proposes the Brain-Gut-Algorithm Axis as a novel explanatory framework. While this review-based analysis synthesizes current evidence to map the scope of this digital crisis, future original quantitative studies are imperative to statistically measure the exact epidemiological damage caused by these short videos. These interventions are essential for reducing health disparities and safeguarding vulnerable populations. Ultimately, reclaiming the epistemic authority of gastroenterology in the digital age demands a unified effort to ensure that technology serves as an instrument of healing rather than a vector for harm.

## Data Availability

The original contributions presented in the study are included in the article/supplementary material, further inquiries can be directed to the corresponding author/s.
